# Flipping it online: re-imagining teaching search skills for knowledge syntheses

**DOI:** 10.29173/jchla29492

**Published:** 2021-08-01

**Authors:** Kaitlin Fuller, Mikaela Gray, Glyneva Bradley-Ridout, Erica Nekolaichuk

**Affiliations:** 1 Liaison & Education Librarian, Gerstein Science Information Centre, University of Toronto Libraries, Toronto, ON; 2 Faculty Liaison & Instruction Librarian, Gerstein Science Information Centre, University of Toronto Libraries, Toronto, ON

## Abstract

**Introduction:**

This program description outlines our approach to re-developing our three-part series for graduate students on comprehensive searching for knowledge syntheses from in-person to online delivery using a flipped classroom model. The re-development coincided with our library’s response to COVID-19.

**Description:**

This series followed a flipped classroom model where participants completed asynchronous modules built on Articulate Rise 360 before attending a synchronous session. Each week of content covered unique learning objectives. Pre- and post-class self-assessments were used to examine students’ understanding of the materials.

**Outcomes:**

152 unique participants registered for the series across two offerings in summer 2020. We observed high engagement with pre-work modules and active participation during synchronous sessions.

**Discussion:**

We found the flipped classroom approach to work well for our users in an online environment. Moving forward, we intend to continue with our re-developed online workshop series with minor modifications, in addition to in-person instruction.

## Introduction

At the University of Toronto, we have been offering a three-part series entitled ‘Strategies for Systematic, Scoping, or Other Comprehensive Searches of Literature’ in-person since 2017 [1]. Over this time, requests to offer the series in an online format were received, however due to librarian capacity, we were unable to create and offer the series through an online format until spring 2020. As laboratory and clinical research was shut down due to precautions around the COVID-19 pandemic, we observed a growing need for review-based research support, which translated to a heightened demand for online educational offerings. This situation was the catalyst for re-developing this workshop series for an online environment. Our goal was to invest the time necessary to develop a program that was purpose-built for online; one that would meet our students’ needs, work well with the learning objectives, and that we could continue to offer in the long-term, even after the COVID-19 pandemic.

Our in-person series includes 7.5 hours of classroom instruction, and previous pedagogy on online teaching has indicated that long durations of screen time are exhausting for students and hinder their ability to absorb content [2,3]. It has also been shown to be less effective to attempt to replicate in-person teaching, no matter how successful, in an online environment [4]. Therefore, while re-imagining this workshop for an online environment, we opted to use a flipped model to provide students with more autonomy and prevent screen fatigue. The flipped classroom model has students complete individual self-paced learning in advance, which is then reinforced through instructor-facilitated group sessions [5]. Previous research has indicated that a flipped classroom approach may work well for information literacy instruction, although challenges, including motivation to complete pre-work, have been reported [6-8]. An online flipped classroom model has also been utilized by other programs as an alternative mode of delivery during the COVID-19 pandemic specifically [9].

This program description will outline our approach to instructional design for this series, including content development, delivery, participation, and instructor reflections.

## Description

The three-part online series was delivered in both June and July 2020. The target audience was graduate students from the health sciences conducting searches for knowledge synthesis (KS) projects, although any member of the University of Toronto community could register.

### Technological Platforms

A working group of four librarians and a library school student adapted content from the in-person series [1]. Multiple tools and platforms were used to create asynchronous modules, manage registration, and facilitate synchronous sessions.

**Fig. 1:**
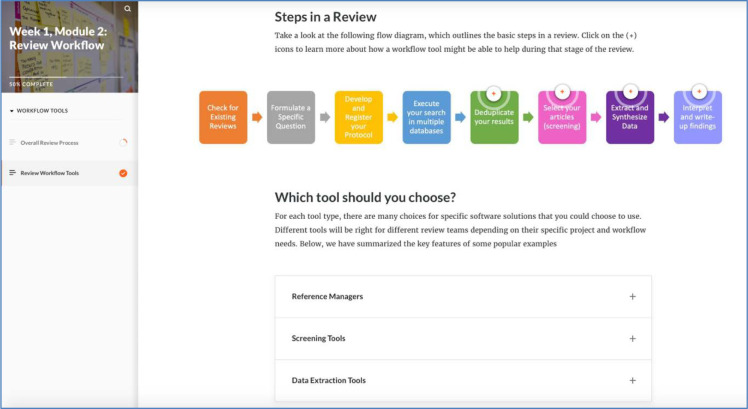
Screenshot of a section of an Articulate Rise 360 module

Over a five-week period, the working group created seven interactive asynchronous modules for the pre-work component. Figure 1 illustrates an example of a module page. Each module was shown to the student in our working group for content suggestions and then piloted by another student. We also consulted with librarians from the University of Calgary who run a similar systematic review workshop series and had recently adapted their content for online [10].

**Fig. 2:**
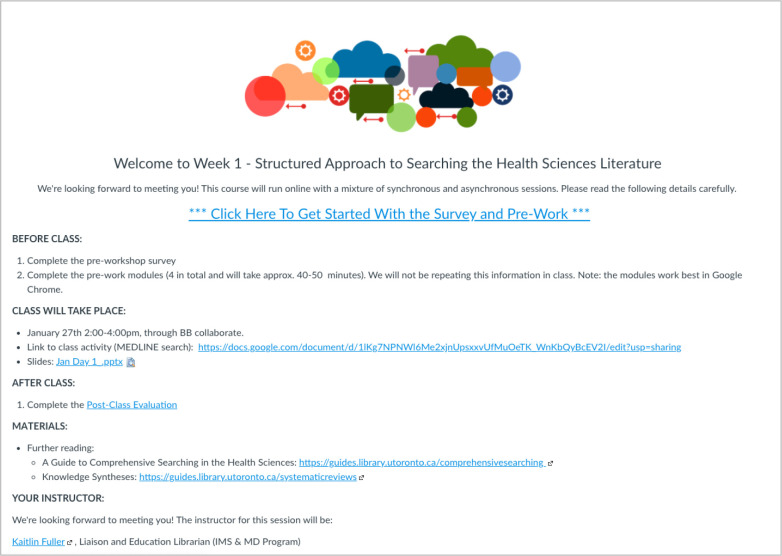
Screenshot of the homepage of the Part 1 Quercus course

Articulate Rise 360 was selected to develop the asynchronous modules after a 30-day free trial period. We selected this tool because of its ease of use, appealing layout, ability to embed interactivity, multiple export options, and the ability to collaborate online. A limitation of Articulate Rise 360 is that knowledge checking data, such as answers to multiple choice questions, is not available. However, this limitation was acceptable in our context as we did not need to evaluate participants. Another limitation of Articulate Rise 360 is that a separate platform, such as a webpage or learning management system (LMS), is needed to host the modules. We selected our institutional LMS, Quercus, which provided students with access to all educational materials (slides, pre- and post-class self-assessments, and downloadable PDF versions of the modules) in one place. Quercus also allowed us to offer the synchronous sessions using the built-in webinar tool Blackboard Collaborate and was able to provide click count data to show how participants were interacting with the modules. Figure 2 displays a screenshot of the organization of our Part 1 Quercus course.

We created pre- and post-class self-assessments (see online supplement, appendix 1) in Quercus for participants to reflect on their attitudes and their learning. These questions were inspired by questions used in the SYstematic Review Center for Laboratory animal Experimentation (SYRCLE) e-learning module [11]. The post-class self-assessment contained the same questions as the pre-class self-assessment with additional questions asking students to reflect on what content was still confusing and what made sense. We also asked for general feedback on the format and delivery in the post-class self-assessment (see online supplement, appendix 1). Furthermore, participants taking the series as part of the Graduate Professional Skills (GPS) program were asked to submit a three-question reflection following the completion of the series (see online supplement, appendix 2).

Online Supplement Appendix 1

Online Supplement Appendix 2

The workshop series was promoted in two primary ways: outreach to liaison areas and through library social media. Registration for the workshop series was managed through LibCal. Each week required separate registration as taking all three sessions was not compulsory. Through reminder emails and workshop descriptions, we communicated that registrants would be expected to review the pre-work modules and be active and engaged during the synchronous sessions.

### Week-by-Week Delivery of Content

The synchronous sessions for all three weeks were delivered by three librarians and supported by a library student. Each synchronous session was two hours long with a 10-minute break. While we considered removing the break to gain additional lecture time, we decided it was important to keep after receiving feedback from our student team member. Our learning objectives for all three weeks remained the same as our in-person offering and can be found in the Online Supplement, Appendix 3 [1]. The exercises, activities, and demonstrations were all based on predetermined examples, but students were encouraged to ask questions about how the examples discussed related to their own research topics.

Online Supplement Appendix 3

## Part I: structured approach to searching the medical literature for knowledge syntheses

The first week of our series focused on describing the differences in review types, identifying workflow tools and resources, and developing an objective, structured method for comprehensive search strategies.

### Pre-Work

The pre-class self-assessment was designed to gauge participants’ understanding of the content. This was followed by three modules covering topics such as KS types, steps to accomplishing a KS, workflow tools, and question creation.

### Synchronous Session

**Fig. 3:**
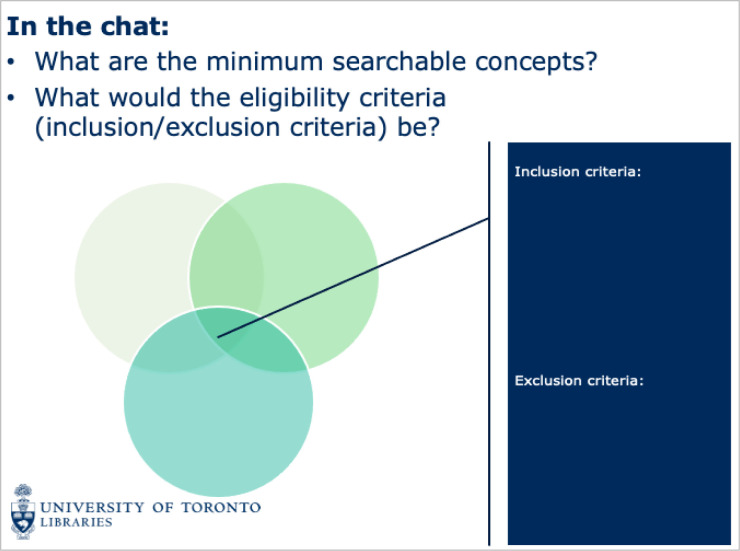
Example of chat box-based activity used in Part 1

To give participants the opportunity to discuss topics covered in the modules, we began with a scenario and an example research question. We then asked a series of questions to generate discussion in the chat box. We also used the chat box to facilitate an activity about search question operationalization (Figure 3). We encouraged additional interaction by using the function in Blackboard Collaborate that allows participants to draw on slides. Breakout groups were used to facilitate an in-depth demonstration of searching in Ovid MEDLINE, where the librarian lead for each group demonstrated the first concept and then prompted students to guide them to complete the second concept. Lastly, videos of how to create a structured search in Ovid MEDLINE were posted on Quercus for future reference.

## Part II: Beyond MEDLINE, translating search strategies for knowledge syntheses

The second week of our series focused on why and how we translate search strategies, including reviewing the Ovid MEDLINE strategy from the first week, diving deeper into advanced features of interfaces and databases, and executing a search translation in OVID Embase and EBSCO CINAHL.

### Pre-Work

Participants were required to complete the pre-class self-assessment and one module, which introduced them to how to search Ovid Embase, EBSCO CINAHL, and CENTRAL on Wiley. These databases were chosen based on their frequency of use in health sciences discipline reviews.

### Synchronous Session

First, we covered additional tips and tricks for developing an Ovid MEDLINE search, including test sets, text mining, and peer review. This was followed by lectures and discussion on the importance of searching in multiple databases. Finally, we demonstrated database translation into OVID Embase and EBSCO CINAHL. This activity involved a librarian leading a demonstration for the first concept and the participants completing the translations in breakout room groups with a Google Doc to facilitate the process. We did not demonstrate CENTRAL on Wiley due to sufficient coverage in the pre-work and limited time in the synchronous session.

## Part III: Going grey and supplementary search techniques

The final week of our workshop series shifted the learners’ focus to searching for grey literature and supplementary searching. We focused on sources of bias, planning a grey literature search, best practices for supplementary searching, and evaluating the search methods in published reviews.

### Pre-Work

Participants were assigned three modules to complete, as well as the pre-class self-assessment. The module topics were reporting bias, grey literature, and supplementary search strategies.

### Synchronous Session

**Fig. 4:**
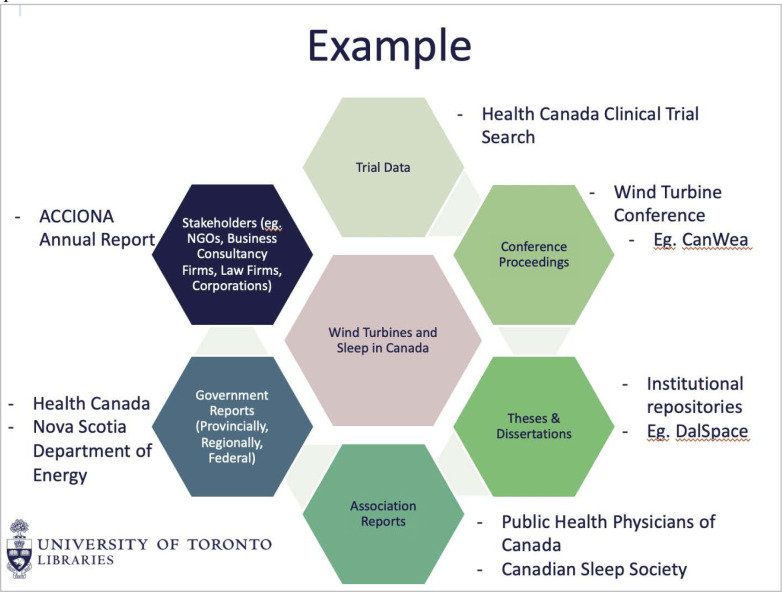
Grey Literature Activity used in Part 3

This session, when compared to the previous two, required the most active involvement from participants. It involved two large activities facilitated in breakout rooms, as well as multiple activities using the chat box, including a scenario that prompted participants to identify opportunities for bias and strategies for addressing bias. Lecture content was minimal and focused on the importance of supplementary searching, reporting search methods, and identifying grey literature needs including where to look and documentation strategies. The first breakout room activity had participants work through the development of a grey literature search plan using a graphic created in Google Slides (Figure 4). The second breakout room activity was a search strategy critical appraisal, guided by probing questions intended to deepen participants’ understanding of the role a search strategy plays in overall review quality

## Outcomes

Registration for the June 2020 online workshop series opened on May 29, 2020 and was filled to capacity at 55 spots within 48 hours. This demonstrated a clear, time-sensitive need from our users. As a result, we opted to schedule a second offering in July 2020, for which registration filled up over a month in advance. The size of our institution, number of health science programs, and the spike in review projects during COVID-19 likely played a role in this demand.

Including the waitlist, this series had 152 unique participants register for the June and July offerings. 55 participants per week were added to the Quercus course, with approximately 37 participants in attendance for each of the synchronous sessions. Due to capacity restrictions, those on the waitlist were only able to participate if space became available. We did not see a higher attrition rate for the online synchronous session when compared with the in-person series, and the majority of the participants were the same for all three sessions. The session where we noted the most variation in participants was Part 3. Although we always encourage participants to take all three sessions, we have had feedback that some participants find it valuable to focus on specific content and customize their learning experience.

We also observed high engagement with our asynchronous content in Quercus. We were able to see both the number of clicks within the course as well as the amount of time each user was active. The interaction counts the week before the first session showed a total of 2,266 engagements. Our highest interaction count from a single day showed 918 engagements from 41 users. Engagements capture the total number of clicks and are not reflective of unique users. The available data indicated that participants were actively engaging with the asynchronous modules prior to attending the synchronous sessions. This allowed us to facilitate fulsome discussions and reinforce and scaffold learning during the synchronous sessions. There was a clear increase in interaction with asynchronous content immediately following reminder emails sent to participants through LibCal.

High levels of engagement during the synchronous sessions were also evident. For example, participants interacted with the instructors, the content, and each other frequently. Participation in the chat box was observed, both during the activities and through questions from participants. Active engagement was also seen during breakout room activities as instructors visited each group. During activities that utilized Google Docs, the instructors were also able to observe participation by viewing the participants’ responses as they worked.

We received a high response rate for our pre- and post-class self-assessments. A total of 224 responses were received for the pre-class self-assessments and 90 responses for the post-class self-assessments. The majority of feedback received in the post-class self-assessment about the format and delivery was positive. Participants cited benefits of the flipped model such as the ability to learn at their own pace, the option to review the content later, and the overall appearance of the Articulate Rise 360 modules. Several participants suggested aspects of the synchronous session that could be added to the pre-work modules going forward. Overall, participants were satisfied with the online environment and found the synchronous sessions complemented the pre-work modules. We received mixed response towards the use of breakout room activities, with some participants enjoying them and others finding collaboration in this medium challenging. In response, we modified our breakout room activities to begin with a prompt for groups to unmute and introduce themselves.

Any participants eligible for GPS credit were required to submit a short reflection-based assignment designed by the original series instructors. A total of twelve GPS reflections were received. The responses were rich and indicated an overall understanding of and critical reflection on the series’ learning objectives. Participants also surfaced and discussed poignant questions such as how to strike the right balance between precision and sensitivity, and how to address the balance between feasibility and rigour.

As with our in-person offerings of this series, we have found our observations and conversations with participants to be the most valuable method of assessment [1]. Observed engagement with asynchronous modules, low attrition, and active participation and engagement in the synchronous sessions indicate that the flipped classroom model was an effective method for delivering this content for our community.

## Discussion

Learning the search skills required for a KS is challenging. We found the flipped model was effective for teaching these skills in an online environment. Students are entering the series from a variety of backgrounds, so the flexibility of the flipped model allowed them to engage with materials at their own pace before participating in the online synchronous session. Active learning is integral to the success of our in-person workshop series, and the flipped approach enabled us to continue to focus on this during the synchronous sessions online. These positive experiences echo previous research on using a flipped classroom model for information literacy instruction, which reported increases in student participation, students feeling empowered when learning, and more opportunities to incorporate active learning techniques [12–14].

Through the design and implementation of this series, we also faced several challenges. Using a flipped classroom model online can add a level of complication to the process of offering an open workshop. Previous research has shown that students are less likely to attend a synchronous session online if they have already been exposed to asynchronous materials compared to in-person and that technological barriers can hinder students from fully participating [15,16]. However, this was not our experience as we did not observe higher attrition rates and did not have any significant technological issues. Instead, we found our challenges were not a result of the online environment, but rather tied to the subject matter in general. For example, the challenge of balancing the critical thinking skills needed to select the appropriate review type and plan for a KS search with the database demonstrations. As with our in-person offerings, we are continually learning how to adapt our materials as new methodologies emerge and as the KS landscape becomes increasingly multidisciplinary.

Another challenge we experienced is that despite the addition of the pre-work modules, we were still very tight for time during the synchronous sessions. We also found that offering this series in an online format was more demanding on our time because of the effort required to develop materials as well as the heavy administrative burden of managing an online course. In addition, “Zoom fatigue” in participants has been documented in the literature. After teaching online, we noticed similar fatigue in ourselves as the instructors [3]. We also observed limited opportunity to build off of participants’ energy. Furthermore, we noted that an increased effort to maintain students’ attention was needed, which has also been reported in previous research on online teaching [17].

We have several next steps for this initiative. We are working on better time management strategies during the synchronous sessions, including how to handle questions more efficiently. We plan to change our lesson plans and reorganize our content to include more pre-work modules. For example, we will move documenting and reporting search strategies, originally covered in lecture format during Part 3, to a pre-work module. We are also planning to rethink our database translation content for Part 2 as we would like to encourage more of a discussion about database selection and the relationship between translation and review types. Overall, we continue to consider how much pre-work participants should be expected to do, how long our synchronous sessions should be, and how to balance the high demand for the series with our own capacities as instructors.

We invested the time and effort necessary to develop this content for an online environment and to ensure our finished product was built to last. We chose the flipped model purposely to allow participants to learn at their own pace and explore topics further through discussions and group activities. We plan to continue to offer this series through a flipped model online in conjunction with the in-person series in the future. We are happy to share educational materials, such as slides, activities, and the Articulate Rise 360 modules. Please contact the authors for access.
