# Supplementary material for: Flipping it online: re-imagining teaching search skills for knowledge syntheses
Source: J Can Health Libr Assoc. 2021 Aug 1;42(2):100–9. doi: 10.29173/jchla29492 (PMC9327592; doi:10.29173/jchla29492)
Supplement: Supplementary file 1 — Online Supplement Appendix 1 [file JCHLA-42-100-s001.pdf]

**Appendix 1:** Pre and post-class self-assessment questions for weeks 1, 2, and 3

Pre-class self-assessment questions, week 1:

1. Why are you taking this workshop? (eg. working on a systematic, scoping or narrative review?).
2. I can clearly define systematic reviews, scoping reviews, narrative reviews, and meta-analyses. *Agree, Disagree, Don't Know*
3. I have a clear understanding of where to find and how to use conduct guidance and reporting guidelines. *Agree, Disagree, Don't Know*
4. A higher proportion of relevant results compared to irrelevant results is a good thing when creating a search for a knowledge synthesis. *Agree, Disagree, Don't Know*
5. Minimum search concepts for a knowledge synthesis typically consist of the population/disease of interest, intervention/exposure, and outcome. *Agree, Disagree, Don't Know*
6. I have a good understanding of what textwords and subject headings are, and how to use them when creating a search in Ovid MEDLINE. *Agree, Disagree, Don't Know*

Post-class self-assessment questions, week 1:

1. What clicked? (an 'Ah-ha'! moment)
2. What's still confusing? (a muddy point)
3. I can clearly define systematic reviews, scoping reviews, narrative reviews, and meta-analyses. *Agree, Disagree, Don't Know*
4. I have a clear understanding of where to find and how to use conduct guidance and reporting guidelines. *Agree, Disagree, Don't Know*
5. A higher proportion of relevant results compared to irrelevant results is a good thing when creating a search for a knowledge synthesis. *Agree, Disagree, Don't Know*
6. Minimum search concepts for a knowledge synthesis typically consist of the population/disease of interest, intervention/exposure, and outcome. *Agree, Disagree, Don't Know*

7. I have a good understanding of what textwords and subject headings are, and how to use them when creating a search in Ovid MEDLINE. *Agree, Disagree, Don’t Know*
8. Offering this workshop online is new for us. Please tell us about your learning experience. What worked for you and what did not work? (eg. what should we keep doing and what should we stop doing)

Pre-class self-assessment questions, week 2:

1. Which one of the below would you like to cover if we have time to review 3 databases in class? *Cochrane CENTRAL (Database of RCTs), Sociological Abstracts (on ProQuest).*
2. If I conduct a sensitive search in MEDLINE, I do not need to search in other subject databases. *Agree, Disagree, Don’t Know*
3. The only reason we search more than 1 database is for unique content. *Agree, Disagree, Don’t Know*
4. I understand how to prepare search methods to ensure proper reporting. *Agree, Disagree, Don’t Know*

Post-class self-assessment questions, week 2:

1. What clicked? (an ‘Ah-ha’! moment)
2. What’s still confusing? (a muddy point)
3. If I conduct a sensitive search in MEDLINE, I do not need to search in other subject databases. *Agree, Disagree, Don’t Know*
4. The only reason we search more than 1 database is for unique content. *Agree, Disagree, Don’t Know*
5. I understand how to prepare search methods to ensure proper reporting. *Agree, Disagree, Don’t Know*
6. Offering this workshop online is new for us. Please tell us about your learning experience. What worked for you and what did not work? (eg. what should we keep doing and what should we stop doing)

Pre-class self-assessment questions, week 3:

1. Reporting bias (such as publication or time lag bias) can impact the data collection for a knowledge synthesis project.
2. I understand what is meant by 'grey literature'.
3. I feel confident in my ability to plan and conduct a grey literature search for a knowledge synthesis
4. Supplementary searches (such as reference tracking/citation searches) are recommended strategies for knowledge synthesis projects

Post-class self-assessment questions, week 3:

1. What clicked? (an ‘Ah-ha’! moment)
2. What’s still confusing? (a muddy point)
3. I understand what is meant by 'grey literature'.
4. I feel confident in my ability to plan and conduct a grey literature search for a knowledge synthesis
5. Supplementary searches (such as reference tracking/citation searches) are recommended strategies for knowledge synthesis projects
6. Offering this workshop online is new for us. Please tell us about your learning experience. What worked for you and what did not work? (eg. what should we keep doing and what should we stop doing)
