# Supplementary material for: Flipping it online: re-imagining teaching search skills for knowledge syntheses
Source: J Can Health Libr Assoc. 2021 Aug 1;42(2):100–9. doi: 10.29173/jchla29492 (PMC9327592; doi:10.29173/jchla29492)
Supplement: Supplementary file 2 — Online Supplement Appendix 2 [file JCHLA-42-100-s002.pdf]

Appendix 2: GPS reflection questions

1. Can better searches improve the quality of research? If yes—how? If no—why?
2. How will you ensure your searches are reproducible and exhaustive?
3. What question(s) has this workshop raised for you?
