# Supplementary material for: Flipping it online: re-imagining teaching search skills for knowledge syntheses
Source: J Can Health Libr Assoc. 2021 Aug 1;42(2):100–9. doi: 10.29173/jchla29492 (PMC9327592; doi:10.29173/jchla29492)
Supplement: Supplementary file 3 — Online Supplement Appendix 3 [file JCHLA-42-100-s003.pdf]

Appendix 3: Learning objectives for each week of the series

Part I: Structured Approach to Searching the Medical Literature for Knowledge Syntheses

1. Identify the key differences between systematic reviews, scoping reviews, and literature reviews
2. Incorporate tools and resources for proper reporting and management of their review
3. Turn a research question into a searchable question
4. Identify databases for their review and explain when to use them
5. Practice using an objective, structured method for developing sensitive search strategies required for knowledge synthesis
6. Apply a structured approach to searching their question in OVID Medline

Part II: Beyond MEDLINE, Translating Search Strategies for Knowledge Syntheses

1. Delve deeper into the advanced features of interfaces and databases which allow for editing and refining a search strategy
2. Translate and execute structured search strategies using different databases, including OVID Embase, and Ebsco CINAHL, CENTRAL on Wiley (if we have time!)
3. Prepare database search strategies and compose search methods, such that they can be repeated and to ensure proper reporting

Part III: Going Grey and Supplementary Search Techniques

1. Identify potential sources for bias in the search and develop strategies to mitigate them
2. Define what grey literature is (and what it is not)
3. Develop a strategy for identifying appropriate sources of grey literature
4. Utilize a methodological, transparent approach to searching sources of grey literature
5. Demonstrate best practices for supplementary search techniques including hand-searching and reference tracking
6. Integrate strategies for incorporating grey literature and supplementary search techniques into the review workflow
7. Evaluate search methods to identify proper reporting
